# Why Is the UAG (Amber) Stop Codon Almost Absent in Highly Expressed Bacterial Genes?

**DOI:** 10.3390/life12030431

**Published:** 2022-03-16

**Authors:** Dominique Belin, Pere Puigbò

**Affiliations:** 1 Department of Pathology and Immunology, University of Geneva, CH1201 Geneva, Switzerland; 2 Department of Biology, University of Turku, 20500 Turku, Finland; 3 Department of Biochemistry and Biotechnology, Rovira i Virgili University, 43007 Tarragona, Catalonia, Spain; 4 Nutrition and Health Unit, Eurecat Technology Centre of Catalonia, 43204 Reus, Catalonia, Spain

**Keywords:** gene, genome, codon usage, genome hypothesis, stop codons, bacterial genomes, highly expressed genes, translational selection, non-sense, translation termination, codon prevalence

## Abstract

The genome hypothesis postulates that genes in a genome tend to conform to their species’ usage of the codon catalog and the GC content of the DNA. Thus, codon frequencies differ across organisms, including the three termination codons in the standard genetic code. Here, we analyze the frequencies of stop codons in a group of highly expressed genes from 196 prokaryotes under strong translational selection. The occurrence of the three translation termination codons is highly biased, with UAA (ochre) being the most prevalent in almost all bacteria. In contrast, UAG (amber) is the least frequent termination codon, e.g., only 321 occurrences (7.4%) in *E. coli* K-12 substr. W3110. Of the 253 highly expressed genes, only two end with an UAG codon. The strength of the selective bias against UAG in highly expressed genes varies among bacterial genomes, but it is not affected by the GC content of these genomes. In contrast, increased GC content results in a decrease in UAA abundance with a concomitant increase in UGA abundance. We propose that readthrough efficiency and context effects could explain the prevalence of UAA over UAG, particularly in highly expressed genes. Findings from this communication can be utilized for the optimization of gene expression.

## 1. Introduction

The degeneracy of the standard genetic code was postulated by Crick et al. in 1961 [1]. In the standard genetic code, most amino acids are indeed encoded by two or more codons, with only methionine and tryptophan having a single codon. Synonymous codons are not used at the same frequency, and this frequency differs in different organisms [2]. Cases of extreme codon usage biases can be found in prokaryotes from low to high guanine + cytosine (GC) content, e.g., in *Escherichia coli* K-12 substr. W3110 (GC = 51.93%), the arginine AGG codon is 20 times less frequent than the CGC one; in *Pseudomonas aeruginosa* PAO1 (GC = 67.14%), the arginine AGG codon is 100 times less frequent than the CGC one; in *Staphylococcus aureus* subsp. *aureus* Mu50 (GC = 33.54%), the proline CCA codon is 15 times less frequent than the CCC one [3]. The differences in codon usage are more evident in ribosomal protein genes and other highly expressed genes (HEG) [4], especially in genomes under strong translational selection [5,6]. 

The three termination codons, UAG (amber), UAA (ochre), and UGA (opal), were first identified as nonsense codons that interrupt translation. The identification of UAG nonsense mutations was greatly facilitated by the frequent occurrence of amber suppressors in commonly used *E. coli* K-12 strains [7,8,9,10]. Furthermore, the amber suppressors are usually highly efficient, so that up to 70% of the amount of the wild-type protein can be produced. UAA (ochre) non-sense mutants and their suppressors were then rapidly identified [11]. In contrast to amber suppressors, ochre suppressors are much less efficient, and ochre mutants cannot be isolated in genes expressed in high amounts, such as phage structural genes. UGA (opal) non-sense mutants and their suppressors were identified later [12,13]. A few years ago, non-sense suppressors were identified in *Haloferax volcanii* [14], thus this is a common mechanism in all domains of life (archaea, bacteria, and eukaryotes). Readthrough of stop codons, whether as an adaptive mechanism or not [15], has been largely described in eukaryotes and bacteria [16]. 

Differential use of the codon repertoire in bacteria [2] is utilized in heterologous gene expression experiments [5]. However, the frequency of stop codons and the following downstream nucleotides has been largely neglected in such experiments. Here, we report the first analysis of the frequency of stop codons and their context in a set of HEG from 196 prokaryotes. We show the three termination codons are also not used at the same frequencies in HEG [17,18]. The UAG stop codon is universally suboptimal in bacteria [17], even more so in HEG. In contrast, UAA is dominant in low and intermediate GC content genomes, while UGA becomes prevalent in high GC content genomes. Moreover, noticeable tendencies in the downstream context arise from the dataset.

## 2. Materials and Methods

### 2.1. Data

A list of predicted highly expressed genes in 196 prokaryotes was gathered from the HEG-DB [6]. This database contains predictions of highly expressed genes in genomes under strong translational selection [5,6]. The *E*. *coli* genes information was obtained from Ecocyc: https://ecocyc.org/ [19], last accessed on 1 March 2022.

### 2.2. Frequency of Stop Codons 

The frequency of stop codons in the list of prokaryotes under translation selection was calculated from the dataset (Table S1). In the genetic code 11 (the bacterial, archaeal, and plant plastid code), the stop codons are UAA, UAG, and UGA. Frequency of stop codons in *E. coli* strain K-12 substr. W3110 was obtained from the codon usage database [3].

### 2.3. Statistics

A Pearson correlation coefficient between frequencies of stop codons and GC content has been calculated using the statistical software package R (https://www.r-project.org/), last accessed on 1 March 2022. Differences in the frequency of +4 nucleotides have been analyzed with a Student’s *t*-test (*p* < 0.01).

## 3. Results and Discussion

**Figure 1:**
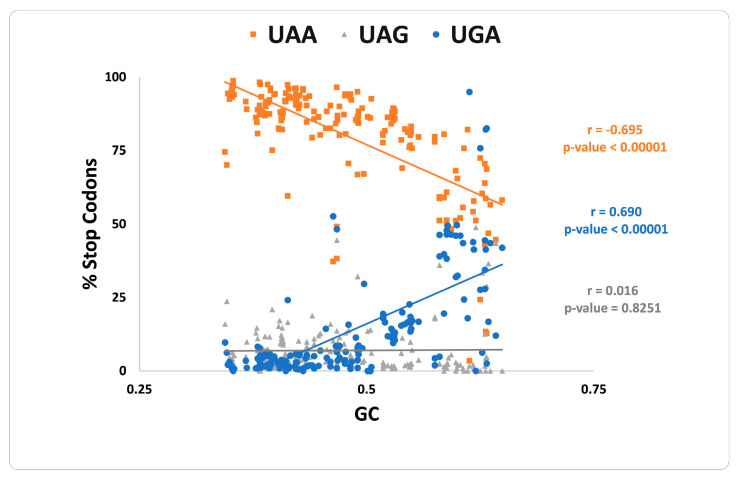
Correlation of the frequency of the three termination codons and the guanine + cytosine (GC) content in the highly expressed genes of 196 prokaryotes under strong translational selection.

In the group of highly expressed genes, an increase in GC content is strongly associated with a parallel increase in UGA and a decrease in UAA content, whereas UAG abundance remains low and largely unchanged (Figure 1), in agreement with previous studies [17,18]. The GC content is a major driver of UGA and UAA stop codons, but its contribution differs between bacteria [20] and archaea [18], and this trend is even stronger in HEG from genomes under strong translational selection. The HEG of 196 prokaryotic species analyzed here includes 192 bacteria (9 actinobacteria, 6 bacteroidetes, 1 deinococcus, 69 firmicutes, and 107 proteobacteria) and 4 archaea (4 euryarchaeota). Among this set, only 18 have more than 15% HEG that terminates with UAG, while this value is less than 5% for more than 100 species. The underrepresentation of the UAG stop codon in HEG is widespread across diverse taxonomic groups (with a few exceptions such as 8/107 proteobacteria, 3/69 firmicutes, and 6/9 actinobacteria) and is independent of the GC content of the organisms. It will be interesting to determine how the force(s) that contribute to counterselection of UAG termination codons in *E. coli* are less effective in these organisms. 

It was proposed that the high suppression efficiency of UAG amber mutations is somehow related to the rare occurrence of this codon at the end of *E. coli* genes. However, this correlation is unlikely to reflect a causal link for two reasons. First, the standard genetic code cannot have evolved in the presence of classical nonsense suppressors that only appear through selective pressure. Such a selective pressure was provided by an *rpoS* amber mutation in the original K-12 strain [10]. In the related B strain, also extensively used in laboratory conditions, most isolates are devoid of suppressor mutations. Second, 29% of the *E. coli* genes and 12% of the HEGs terminate with a UGA codon, even though suppression of UGA opal mutations occurs with a >50% efficiency. 

In *E. coli*, the three termination codons, UAG, UAA, and UGA, are not used at the same frequencies, with only 321 genes ending with UAG (7.4%), while 2765 (64%) end with UAA and 1249 (29%) end with UGA. With the identification of HEG, this difference becomes even more striking. Out of 253 HEG in *E. coli*, 223 (87%) end with UAA, 30 (12%) with UGA and only 2 (0.8%) with UAG. The two HEG that end with UAG are *atpE* and *sucB*. The *atpE* gene, which is essential in both rich and minimal media, encodes a membrane-embedded subunit of the Fo complex. Its UAG termination codon is immediately followed by a UAA codon. Thus, suppression or read through of the *atpE* UAG would only add one residue at the C-terminus, which faces the cytoplasm. The *sucB* gene, which is only essential in some rich media, encodes a cytoplasmic enzyme involved in lysine degradation that is a component of the 2-oxoglutarate dehydrogenase complex. The UAG termination codon is immediately followed by a second UAG codon, and the next termination codon (UGA) is 21 codons away. In this case, readthrough of both UAG could affect the activity or stability of SucB. Readthrough of nonsense codons refers to continued translation elongation in the absence of a suppressor tRNA. Gln is inserted at both UAG and UAA codons, although termination is much more efficient with UAA (about 0.2%) [21]. In the case of UGA, readthrough occurs with an efficiency of about 2% and results from the incorporation of Trp [13]. 

**Figure 2:**
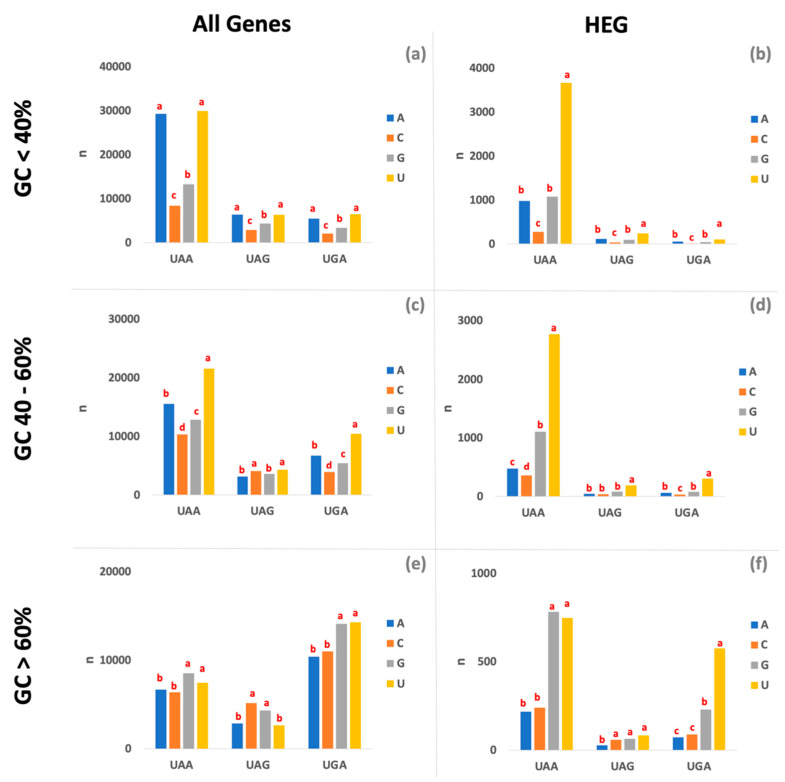
**Frequency of nucleotides after the stop codons.** (**a**) All genes from genomes with a %GC lower than 40% (**b**) HEG from genomes with a %GC lower than 40% (**c**) All genes from genomes with a %GC between 40% and 60% (**d**) HEG from genomes with a %GC between 40% and 60% (**e**) All genes from genomes with a %GC higher than 60% (**f**) HEG from genomes with a %GC higher than 60. Groups within stop codons are determined based on a Student’s t-test (*p* < 0.01).

The termination efficiency of nonsense mutations varies at different sites; this phenomenon is called the context effect. This was first observed with *phoA* UAG mutants in a strain carrying an amber suppressor [8]. Termination could be as high as 97% at one site and as low as <1% at another [8]. Starting with an UAA mutant that was poorly suppressed by an ochre suppressor and thus efficiently terminated, mutations in the immediate vicinity of the UAA codon decreased termination by at least 10-fold [22]. Similar results have been observed with the base (also known as +4 nucleotide) following UGA codons [23]. Termination efficiency was also shown to vary extensively at many sites within the *lacI* gene [24,25]. In general, between one (+4) and six (+9) nucleotides after the stop codon are critical for termination efficiency [16,26,27]. A role for context effects is illustrated by the higher prevalence of the U residues at the 3′ side of UAA and UGA in HEGs when compared to low expressed genes (LEG) [28]. Our results show the prevalence of U nucleotides after the stop codon is dominant independently of the GC content of the genome, especially in the HEG (Figure 2). Although, in some genomic contexts, U residues share dominance with another nucleotide, e.g., in AT-rich genomes, U and A residues are equally frequent after the stop codon. The dominance of U residues after the stop codon is almost universal in HEG, with the exception of UAA-ending genes in GC-rich genomes, where U residues share dominance with G residues. Overall, results indicate a set of rules that largely determine the context effect that drives the efficiency of gene expression (Figure 2). 

**Figure 3:**
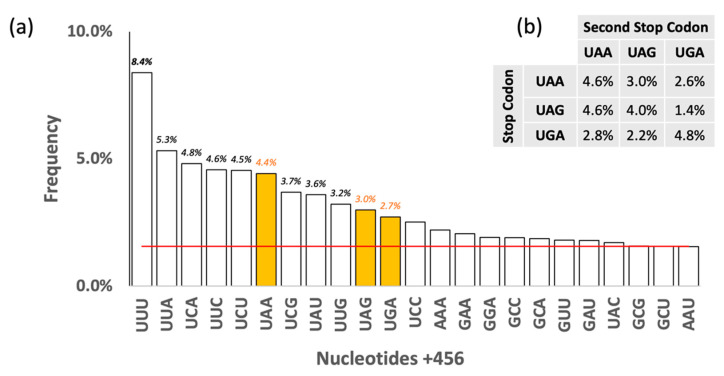
**Frequency of nucleotides after the stop codons (+456) in the HEG.** (**a**) Most frequent codons downstream of the stop codon. The red line indicates chance expectations (1/64 = 2%). Only codons occurring with or above the chance expectation are indicated. (**b**) Frequency of tandem stop codons.

Some authors have debated whether a second stop codon may be present after the termination one to increase termination efficiency [29,30,31,32], as in the two *E. coli* K-12 HEG that end with UAG (*atpE* and *sucB*). However, neither HEG nor LEG have a higher frequency of additional stop codons after the termination one, although there is a dominance of UNN (N = any nucleotide) triplets and a detectable prevalence of UUU (Figure 3). Moreover, it is interesting to notice that 17/18 genomes with more than 15% UAG in their HEG have an extended preference for certain residues for at least 10 residues on the 3′ side of the UAG codon.

Thus, we hypothesize that low readthrough efficiency and context effects may provide a better explanation for the prevalence of UAA termination codons over UAG, particularly in highly expressed genes. Moreover, the wide suppression across taxonomic groups of the UAG stop codon in bacteria is an indication that this is an ancient mechanism. However, a few taxonomically diverse species have higher %UAG than expected, thus we speculate that they may have developed alternative strategies to efficiently overcome readthroughs.

## 4. Conclusions

Our data indicate that UAG codon is universally suboptimal in the HEG from prokaryotes under translational selection. UAA is likely to be the preferred stop codon for low or intermediate GC content, whereas UGA is the preferred stop codon for high GC content. Readthrough efficiency and additional context effects may explain the dominance of UAA and UGA stop codons in HEG. Although the UAA stop codon is usually the choice in genome engineering and to optimize gene expression, the differential use of the stop codons in the HEG and the downstream context described in this article should be taken into consideration.

## Data Availability

The frequencies of stop codons in the predicted highly expressed genes are freely available at https://ppuigbo.me/programs/HEG-DB (Stop Codons).

## Supplementary Materials

The following supporting information can be downloaded at: https://www.mdpi.com/article/10.3390/life12030431/s1, Table S1: Frequency of stop codons and other genomic parameters in 196 prokaryotic genomes under strong translational selection. Abbrev: abbreviation in the HEG-DB, Name: Species name, bp: total number of base pairs, n_genes: number of genes; n_tRNA: number of tRNA; n_RPG: number of ribosomal protein genes; GC: guanine + cytosine content; GC1: guanine + cytosine content at the first codon position; GC2: guanine + cytosine content at the second codon position; GC3: guanine + cytosine content at the third codon position; Stop_Codons: number of stop codons; UAA: number of UAA stop codons, UAG: number of UAG stop codons, UGA: number of UGA stop codons, %UAA: percentage of UAA stop codons; %UAG: percentage of UAG stop codons and %UGA: percentage of UGA stop codons.

## References

[1] Crick F.H., Barnett L., Brenner S., Watts-Tobin R.J. (1961). General nature of the genetic code for proteins. Nature.

[2] Grantham R., Gautier C., Gouy M., Mercier R., Pavé A. (1980). Codon catalog usage and the genome hypothesis. Nucleic Acids Res..

[3] Nakamura Y., Gojobori T., Ikemura T. (2000). Codon usage tabulated from international DNA sequence databases: Status for the year 2000. Nucleic Acids Res..

[4] Sharp P.M., Li W.H. (1987). The codon Adaptation Index--a measure of directional synonymous codon usage bias, and its potential applications. Nucleic Acids Res..

[5] Puigbò P., Guzmán E., Romeu A., Garcia-Vallvé S. (2007). OPTIMIZER: A web server for optimizing the codon usage of DNA sequences. Nucleic Acids Res..

[6] Puigbò P., Romeu A., Garcia-Vallvé S. (2008). HEG-DB: A database of predicted highly expressed genes in prokaryotic complete genomes under translational selection. Nucleic Acids Res..

[7] Benzer S., Champe S.P. (1962). A change from nonsense to sense in the genetic code. Proc. Natl. Acad. Sci. USA.

[8] Garen A., Siddiqi O. (1962). Suppression of mutations in the alkaline phosphatase structural cistron of *E. coli*. Proc. Natl. Acad. Sci. USA.

[9] Brenner S., Stretton A.O., Kaplan S. (1965). Genetic code: The “nonsense” triplets for chain termination and their suppression. Nature.

[10] Belin D. (2003). Why are suppressors of amber mutations so frequent among *Escherichia coli* K12 strains? A plausible explanation for a long-lasting puzzle. Genetics.

[11] Brenner S., Beckwith J.R. (1965). Ochre mutants, a new class of suppressible nonsense mutants. J. Mol. Biol..

[12] Brenner S., Barnett L., Katz E.R., Crick F.H. (1967). UGA: A third nonsense triplet in the genetic code. Nature.

[13] Sambrook J.F., Fan D.P., Brenner S. (1967). A strong suppressor specific for UGA. Nature.

[14] Bhattacharya A., Köhrer C., Mandal D., RajBhandary U.L. (2015). Nonsense suppression in archaea. Proc. Natl. Acad. Sci. USA.

[15] Li C., Zhang J. (2019). Stop-codon read-through arises largely from molecular errors and is generally nonadaptive. PLoS Genet..

[16] Namy O., Hatin I., Rousset J.P. (2001). Impact of the six nucleotides downstream of the stop codon on translation termination. EMBO Rep..

[17] Povolotskaya I.S., Kondrashov F.A., Ledda A., Vlasov P.K. (2012). Stop codons in bacteria are not selectively equivalent. Biol. Direct.

[18] Belinky F., Babenko V.N., Rogozin I.B., Koonin E.V. (2018). Purifying and positive selection in the evolution of stop codons. Sci. Rep..

[19] Keseler I.M., Gama-Castro S., Mackie A., Billington R., Bonavides-Martínez C., Caspi R., Kothari A., Krummenacker M., Midford P.E., Muñiz-Rascado L. (2021). The ecocyc database in 2021. Front. Microbiol..

[20] Korkmaz G., Holm M., Wiens T., Sanyal S. (2014). Comprehensive analysis of stop codon usage in bacteria and its correlation with release factor abundance. J. Biol. Chem..

[21] Nilsson M., Rydén-Aulin M. (2003). Glutamine is incorporated at the nonsense codons UAG and UAA in a suppressor-free Escherichia coli strain. Biochim. Biophys. Acta.

[22] Fluck M.M., Epstein R.H. (1980). Isolation and characterization of context mutations affecting the suppressibility of nonsense mutations. Mol. Gen. Genet..

[23] Engelberg-Kulka H. (1981). UGA suppression by normal tRNA Trp in Escherichia coli: Codon context effects. Nucleic Acids Res..

[24] Miller J.H., Albertini A.M. (1983). Effects of surrounding sequence on the suppression of nonsense codons. J. Mol. Biol..

[25] Bossi L. (1983). Context effects: Translation of UAG codon by suppressor tRNA is affected by the sequence following UAG in the message. J. Mol. Biol..

[26] Poole E.S., Brown C.M., Tate W.P. (1995). The identity of the base following the stop codon determines the efficiency of in vivo translational termination in Escherichia coli. EMBO J..

[27] Tate W.P., Poole E.S., Dalphin M.E., Major L.L., Crawford D.J., Mannering S.A. (1996). The translational stop signal: Codon with a context, or extended factor recognition element?. Biochimie.

[28] Wei Y., Xia X. (2017). The role of +4U as an extended translation termination signal in bacteria. Genetics.

[29] Liang H., Cavalcanti A.R.O., Landweber L.F. (2005). Conservation of tandem stop codons in yeasts. Genome Biol..

[30] Major L.L., Edgar T.D., Yee Yip P., Isaksson L.A., Tate W.P. (2002). Tandem termination signals: Myth or reality?. FEBS Lett..

[31] Ho A.T., Hurst L.D. (2019). In eubacteria, unlike eukaryotes, there is no evidence for selection favouring fail-safe 3′ additional stop codons. PLoS Genet..

[32] Nichols J.L. (1970). Nucleotide sequence from the polypeptide chain termination region of the coat protein cistron in bacteriophage R17 RNA. Nature.

